# 

**Published:** 1903-11-28

**Authors:** 


					THE HOSPITAL. "The Standard of highest purity."?Lancet. November 28th, 1903,
CADBURY'S fcl CADBURY'S
GOCOA JL mm JgL, COCOA
?A PERFECT FOOD." NO DRUGS OR ALKALIS* UUIX
No. 896. Yol. XXXV. [aSPTP3 Saturday,
Nov. 28th, 1903.
[SuhscripUon^Terms,] pRICE THREEpE||CE>

				

